# Novel causative *RYR2* indel variant with exon and intron involvement inducing exon 13 skipping in a family exhibiting catecholaminergic polymorphic ventricular tachycardia 

**DOI:** 10.3389/fgene.2025.1581535

**Published:** 2025-06-18

**Authors:** Ju Hyeon Shin, Taek Kyu Park, Sung-A. Chang, Shin Yi Jang, June Huh, Chang Ahn Seol, Kyoung-Jin Park, Sung Hoon Kim, Duk-Kyung Kim, Hye Bin Gwag, Mi-Ae Jang

**Affiliations:** ^1^ Department of Laboratory Medicine and Genetics, Samsung Medical Center, Sungkyunkwan University School of Medicine, Seoul, Republic of Korea; ^2^ Division of Cardiology, Department of Medicine, Heart Vascular Stroke Institute, Samsung Medical Center, Sungkyunkwan University School of Medicine, Seoul, Republic of Korea; ^3^ Division of Cardiology, Department of Pediatrics, Adult Congenital Heart Disease Clinic, Heart Vascular Stroke Institute, Samsung Medical Center, Sungkyunkwan University School of Medicine, Seoul, Republic of Korea; ^4^ GC Genome, Yongin-si, Gyeonggi-do, Republic of Korea; ^5^ GC Labs, Yongin-si, Gyeonggi-do, Republic of Korea; ^6^ Department of Laboratory Medicine, Samsung Changwon Hospital, Sungkyunkwan University School of Medicine, Changwon-si, Republic of Korea; ^7^ Department of Pediatrics, Samsung Changwon Hospital, Sungkyunkwan University School of Medicine, Changwon-si, Republic of Korea; ^8^ Division of Cardiology, Department of Internal Medicine, Samsung Changwon Hospital, Sungkyunkwan University School of Medicine, Changwon-si, Republic of Korea

**Keywords:** *RYR2*, catecholaminergic polymorphic ventricular tachycardia, RNA splicing, protein structure, variant interpretation

## Abstract

Catecholaminergic polymorphic ventricular tachycardia (CPVT) is a genetic disorder marked by exercise or stress-induced arrhythmias that lead to syncope or sudden cardiac death. Mutations of the *RYR2* gene can cause either CPVT or calcium release deficiency syndrome, with varying impacts on calcium release in cardiomyocytes. These mutations are predominantly missense variants associated with a gain-of-function mechanism. In this report, we present a novel pathogenic *RYR2* indel variant in a family afflicted with CPVT based on comprehensive molecular investigations. The proband was a 15-year-old girl who suffered a cardiac arrest during exercise and exhibited frequent premature ventricular beats on a treadmill test, which was consistent with CPVT. Using next-generation sequencing and Sanger sequencing, a novel *RYR2* indel variant, NM_001035.3:c.1006-44_1007delinsATTTTG, was identified. Sanger sequencing confirmed the presence of this variant in her mother, who also showed frequent premature ventricular beats on a treadmill test. Further RNA analysis revealed that this variant caused aberrant splicing, resulting in the skipping of exon 13 (r.1006_1170del), which would disrupt the intramolecular domain interactions. This discovery led to the classification of the variant as a likely pathogenic variant. We identified a novel *RYR2* indel variant responsible for CPVT and expanded the mutational spectrum of *RYR2*-related CPVT, emphasizing the importance of comprehensive genetic approaches for variant classification.

## 1 Introduction

Catecholaminergic polymorphic ventricular tachycardia (CPVT; MIM 604772) is an inherited arrhythmogenic disorder representing ventricular arrhythmias induced by physical exercise, adrenergic stress, or catecholamine administration (Steinberg et al., 2023). A patient with CPVT has a high probability of recurrent syncope, seizure, cardiac arrest, and sudden cardiac death (Tester et al., 2004; Rucinski et al., 2020; Wilde et al., 2022). CPVT is diagnosed as follows: in an individual under 40 years of age, the presence of a structurally normal heart, normal electrocardiogram (ECG), and unexplained exercise- or catecholamine-induced bidirectional or polymorphic ventricular tachycardia; in an individual who has a pathogenic variant (Priori et al., 2013; Wilde et al., 2022). CPVT is associated with several genes, including *RYR2*, *CASQ2*, *CALM1*, *CALM2*, *CALM3*, *KCNJ2*, *TECRL*, and *TRDN*, where *RYR2*-related CPVT accounts for 60%–70% of all cases (Wilde et al., 2022). Beta-adrenoreceptor blocker therapy is the treatment of choice for CPVT. Occasionally, an implantable cardioverter defibrillator (ICD) is considered for a patient whose symptoms are not controllable using beta-blockers since ICD shocks can increase sympathetic tone and induce a malignant cycle of ICD shocks (Priori et al., 2013; van der Werf et al., 2019; Wilde et al., 2022).

The *RYR2* gene encodes the cardiac ryanodine receptor, which is the main calcium-releasing channel of the sarcoplasmic reticulum in the heart (Steinberg et al., 2023). It is associated with cardiac ryanodinopathies, including CPVT and calcium release deficiency syndrome (CRDS; MIM 115000) in an autosomal dominant inheritance pattern. Pathogenic *RYR2* variants found in individuals with CPVT are associated with a gain-of-function mechanism leading to leakage of calcium into the cytosol (Wilde et al., 2022). By contrast, *RYR2* variants associated with a loss-of-function mechanism cause CRDS, which is characterized by a negative result of the exercise stress test unlike CPVT (Fujii et al., 2017; Sun et al., 2021; Roston et al., 2022; Wilde et al., 2022). Pathogenic *RYR2* variants registered in the Human Gene Mutation Database and ClinVar are mostly missense variants associated with gain-of-function mechanisms (Steinberg et al., 2023). Interestingly, deletion of the *RYR2* exon 3 has been known to disrupt the intramolecular domain interactions, resulting in facilitated pore opening and showing a CPVT-like phenotype (Lobo et al., 2011).

In the present work, we performed genetic investigations of a family with CPVT. In the proband and her mother, a novel *RYR2* indel variant, NM_001035.3:c.1006-44_1007delinsATTTTG, with both exon and intron involvement was detected. This variant removes the 44 intronic nucleotides upstream of exon 13 and the two exonic nucleotides into exon 13, replacing them with six nucleotides (ATTTTG). RNA splicing analysis demonstrated the skipping of exon 13 of the *RYR2* gene, which enabled us to classify the variant as a likely pathogenic variant.

## 2 Methods

### 2.1 Study subjects

Two members of a family affected by CPVT were investigated at both Samsung Changwon Hospital and Samsung Medical Center. Informed consent for genetic testing and research use of the biological data was obtained from all investigated subjects. This study was approved by the institutional review board of Samsung Changwon Hospital (approval no. 2024–09-003).

### 2.2 Panel-based next-generation sequencing (NGS) assay

Genomic DNA was extracted from the peripheral blood leukocytes and underwent panel-based NGS for various genes, including *ANK2*, *CACNA1C*, *CACNA2D1*, *CACNB2*, *CALM1*, *CALM2*, *CALM3*, *CASQ2*, *KCNE1*, *KCNE2*, *KCNH2*, *KCNJ2*, *KCNQ1*, *RYR2*, *SCN5A*, *SLC4A3*, *TANGO2*, *TECRL*, and *TRDN*, which are associated with arrhythmias. A custom panel (IDT, Coralville, IA, United States) was used for the library preparation, and sequencing was performed on the NextSeq platform (Illumina, San Diego, CA, United States). The DNA sequence reads were aligned to reference sequences based on the public human genome build GRCh37/UCSC hg19; alignments were then conducted with BWA-mem (version 0.7.17), and the duplicate reads were marked with biobambam2 and base quality recalibration, followed by variant calling with the Genome Analysis Tool Kit (GATK, version 4.1.8); lastly, annotations were performed using Variant Effect Predictor and dbNSFP. The evaluations were initially focused on coding exons along with their flanking ±20 intronic bases; however, these were extended to the complete gene region for candidate genes or searched for a previously described second variant in an autosomal recessive inheritance pattern. The sequence variants were classified according to the guidelines of the American College of Medical Genetics and Genomics/Association for Molecular Pathology (ACMG/AMP) (Richards et al., 2015).

### 2.3 Sanger sequencing

Sanger sequencing was performed to validate the candidate variants identified in the panel-based NGS assay and to determine whether these variants were present in the family members. Genomic DNA was extracted from the peripheral blood leukocytes, and the targeted exons of *RYR2* were amplified by polymerase chain reaction (PCR) using self-designed primers (Supplementary Table S1). The PCR products were sequenced on an ABI 3730xl DNA Analyzer (Applied Biosystems, Foster City, CA, United States) using a BigDye Terminator Cycle sequencing kit (Applied Biosystems). The sequences were analyzed using Sequencher software (Gene Codes Corp., Ann Arbor, MI, United States) and compared with the reference sequence for *RYR2* (NM_001035.3).

### 2.4 Reverse transcription PCR (RT-PCR) and RNA sequencing

The total RNA was extracted from the lymphocyte fraction isolated from peripheral blood using the TRIzol method. Next, the RNA was reverse-transcribed into cDNA using the Omniscript reverse transcriptase kit (Qiagen, Hilden, Germany) and amplified using Platinum II Hotstartaq DNA polymerase (Thermo Fisher Scientific, Waltham, MA, United States) with specifically designed primers (Supplementary Table S1). In agarose gel electrophoresis of the test samples, we identified any abnormal PCR bands that differed in size from the expected result. For the subsequent Sanger validation, the PCR products were sequenced using the ABI 3730xl DNA Analyzer. The sequences were then analyzed with Sequencher software and compared with the *RYR2* reference sequence (NM_001035.3). Gel images that conformed with the digital image and integrity policies were obtained from parallelly processed samples from the same experiment.

### 2.5 Prediction of alternative splicing and protein structure

Potential splicing of a variant was predicted using SpliceAI (Jaganathan et al., 2019), where delta scores of 0.2 or higher were considered to have deleterious effects on normal splicing. The position of the affected acceptor or donor site was also provided with the delta score. Molecular models of the tertiary structures were generated using AlphaFold 3 model (https://alphafoldserver.com) (accessed on 17 September 2024) developed by Google DeepMind and Isomorphic Labs (Abramson et al., 2024). Images of the predicted proteins of the wild-type and mutant structures were obtained using Mol* 3D viewer (https://www.rcsb.org/3d-view) (Sehnal et al., 2021).

## 3 Results

### 3.1 Clinical investigation

**FIGURE 1:**
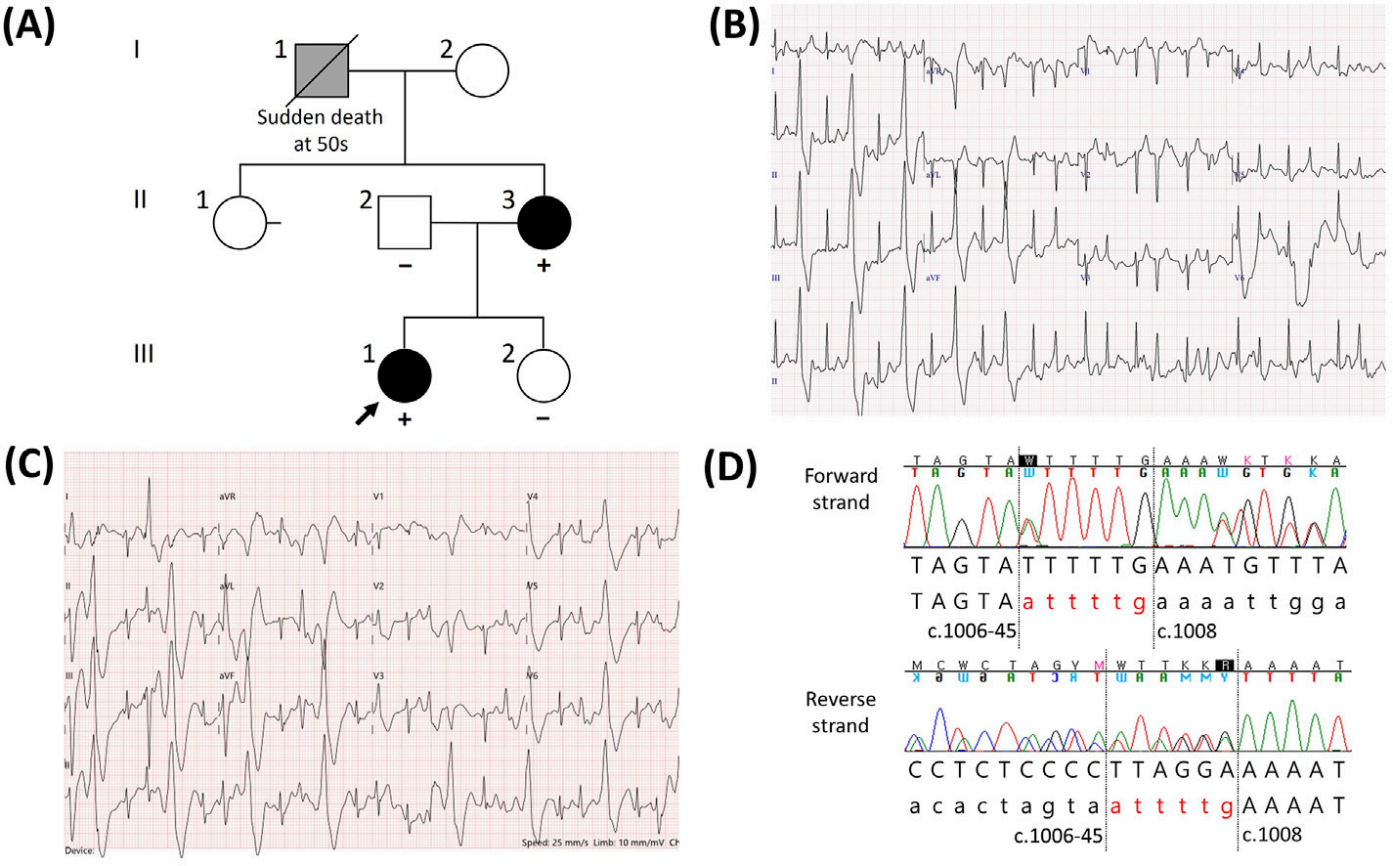
Catecholaminergic polymorphic ventricular tachycardia (CPVT) in a family having the novel *RYR2* indel variant NM_001035.3:c.1006-44_1007delinsATTTTG. **(A)** Pedigree of the family. For individuals who underwent a genetic test for the *RYR2* gene, the results are described with either “+” (variant detected) or “−” (variant not detected). Individuals diagnosed with CPVT are shown in black, whereas individuals who were assumed to suffer from CPVT but not definitively diagnosed are shown in gray. **(B)** Electrocardiogram (ECG) of the proband during a treadmill test showing frequent ventricular premature beats. **(C)** ECG of the proband’s mother during the treadmill test showing frequent ventricular premature beats similar to that of the proband. **(D)** Forward and reverse sequences showing the indel variant NM_001035.3 (*RYR2*):c.1006-44_1007delinsATTTTG. Forty-four intronic nucleotides located upstream of exon 13 and the first two nucleotides of exon 13 were replaced with ATTTTG.

The proband was a 15-year-old girl (III:1, Figure 1A) who underwent aborted cardiac arrest and was admitted to Samsung Changwon Hospital. She was successfully resuscitated using an automated external defibrillator from cardiac arrest during exercise at school. The ECG between the arrest and resuscitation was not obtained at the school, and the initial ECG at the hospital showed normal rhythm. To differentiate the cause of cardiac arrest, a treadmill test and echocardiography were performed. The clinical tests demonstrated frequent premature ventricular beats during the treadmill test (Figure 1B) and a structurally normal heart via echocardiography. The proband’s mother, a 44-year-old woman (II:3, Figure 1A), had no history of syncope and showed no identifiable rhythm of ventricular tachycardia in the resting ECG; however, symptomatic frequent premature ventricular beats were observed during a treadmill test, similar to the proband (Figure 1C). Ventricular tachycardia was not documented for the proband and her mother on a Holter monitor. The proband’s grandfather (I:1, maternal, Figure 1A) was reported to have experienced sudden death from an unknown cause in his fifties; although an autopsy had not been conducted, the tentative diagnosis at that time of death was stroke. The family members did not remember the specific situation when the event occurred. The proband’s father (II:2) and younger sister (III:2) had no similar cardiological symptoms. The proband (III:1) and her mother (II:3) were treated with the beta-adrenoreceptor blocker nadolol, and their symptomatic ventricular premature beats were well controlled.

### 3.2 Genetic investigation

A panel-based NGS assay for genes related to hereditary arrhythmia and subsequent Sanger sequencing were performed on the proband. Excluding the variants that were classified as (likely) benign according to the ACMG/AMP guidelines, two candidate heterozygous variants were detected: NM_001035.3 (*RYR2*):c.1006-44_1007delinsATTTTG (Figure 1D) and NM_001148.6 (*ANK2*):c.11320G>A. Because an *ANK2* variant was found at a frequency of 0.09% in the East Asian population (gnomAD v2.1.1) and was predicted to be benign based on *in silico* analysis (REVEL score of 0.283), we focused on evaluating the *RYR2* indel variant. This indel variant removes the 44 intronic nucleotides upstream of exon 13 as well as two exonic nucleotides into exon 13, replacing them with six nucleotides (ATTTTG). For the family members, we performed targeted Sanger sequencing of the *RYR2* variant. This variant was also detected in the proband’s mother; however, it was not detected in the father or younger sister (Figure 1A). According to the ACMG/AMP guidelines, this variant meets the PM2 (not found in general population databases), PP3 (predicted to be deleterious *in silico*), and PP1 (co-segregation within a family) criteria. However, these criteria were not sufficient to classify the variant as pathogenic, resulting in its classification as a variant of uncertain significance (VUS).

**FIGURE 2:**
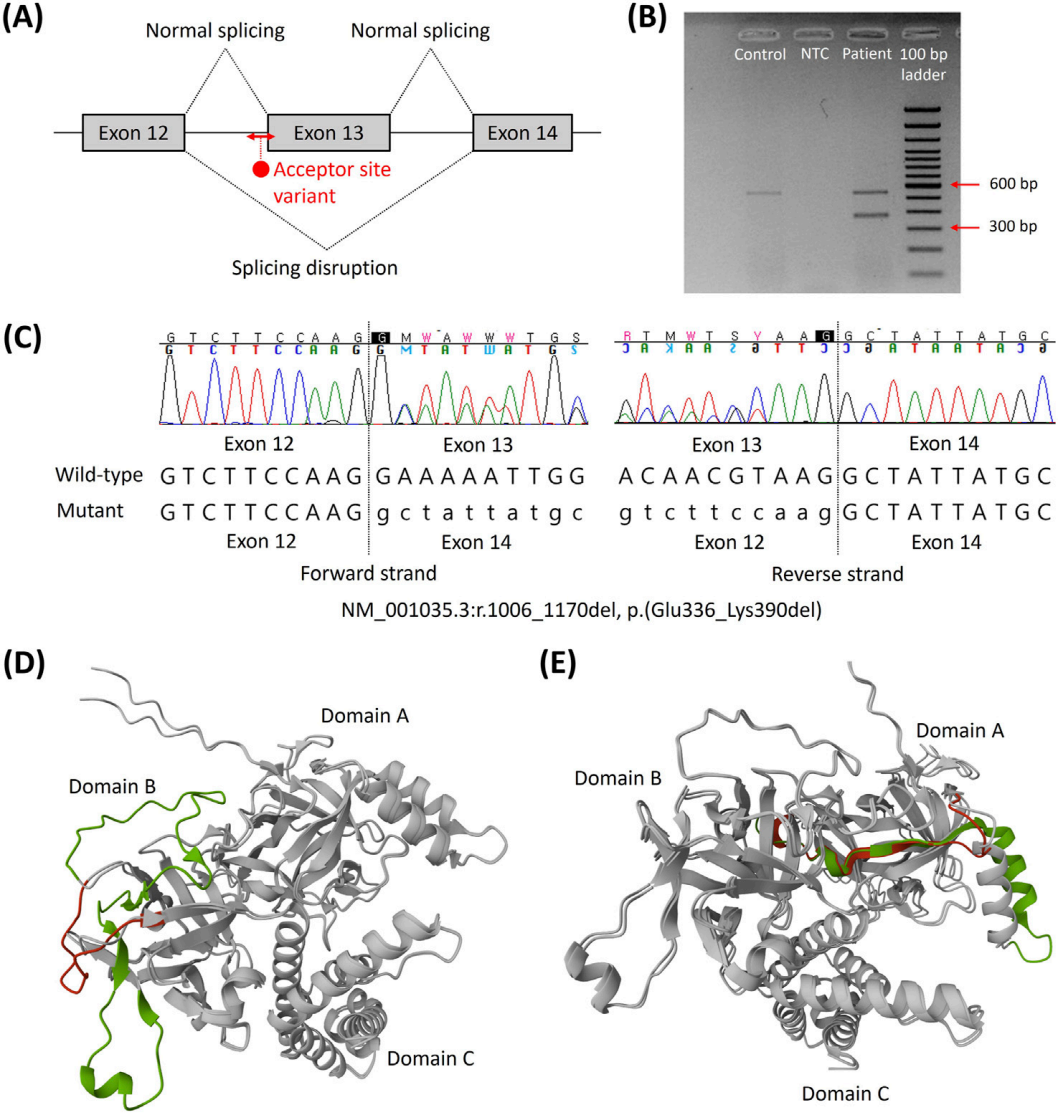
Splicing effects of the novel *RYR2* indel variant NM_001035.3:c.1006-44_1007delinsATTTTG. **(A)** Schematic of the targeted RNA sequencing and cDNA sequence of the aberrant transcript. **(B)** Compared to the control, a shorter band with 165 bp missing was observed. **(C)** Forward and reverse sequences showing the skipping of exon 13, NM_001035.3:r.1006_1170del, which leads to in-frame deletion of 55 amino acids. **(D)** Superposition of the predicted protein structures of the N-terminal domains of the wild type and exon 13 deletion. Exon 13 is shown in green, and the segment changes compared to the wild type are shown in red. **(E)** Superposition of the predicted protein structures of the N-terminal domains of the wild type and exon 3 deletion. Exon 3 and the rescue segment are shown in green and red, respectively (Lobo et al., 2011). Abbreviations: bp, base pair; NTC, no template control.

To elucidate the pathogenicity of this VUS, an additional RNA study was performed at Samsung Medical Center. *In silico* analysis (SpliceAI) predicted the loss of an acceptor splice site at position c.1006 with a high prediction score of 1.00, which would induce exon 13 skipping (Figure 2A). Based on RT-PCR and agarose gel electrophoresis, the wild-type cDNA was observed to have a band of the expected size of 578 bp; however, the mutated cDNA had an additional band that was approximately 165 bp shorter than the wild-type cDNA (Figure 2B). The presence of the abnormal transcript of size 413 bp indicated that the *RYR2* indel variant caused aberrant splicing of the mRNA, resulting in the skipping of exon 13, as predicted by SpliceAI (Figure 2C). The skipping of exon 13 resulted in deletion of the 165 bp (r.1006_1170del), resulting in in-frame deletion of 55 amino acids (p.(Glu336_Lys390del)) instead of disruption of the reading frame. The protein structures of the N-terminal domain (NTD) of the wild-type and mutant genes predicted by AlphaFold 3 demonstrated that the truncating part was adjacent to other tetrameric subunits (Figure 2D). This removes some of the β strands of the NTD (exons 3–15), which is the one of the mutational hotspots of the cardiac ryanodine receptor (Hadiatullah et al., 2022; Steinberg et al., 2023). The PVS1_strong criterion was applied to the variant instead of PP3 based on the results of splicing analysis. Consequently, the *RYR2* indel variant c.1006-44_1007delinsATTTTG was reclassified as a likely pathogenic variant.

## 4 Discussion

Panel-based NGS assay and subsequent splicing analysis were used to establish a suspected genetic cause for CPVT in this family. The *RYR2* indel variant c.1006-44_1007delinsATTTTG detected in this family has not been reported previously; this novel pathogenic variant, which involves both exon 13 and intron 12, was confirmed through RNA study to induce aberrant splicing, resulting in exon 13 skipping. Although *RYR2* is known to be expressed mainly in the cardiac muscle, it is also expressed in various other tissues, including whole blood, according to the GTEx Portal and dbGaP Accession phs000424.v10.p2 (GTEx Consortium, 2013; Hadiatullah et al., 2022). Therefore, RNA was extracted from the peripheral blood lymphocytes to detect aberrant splicing in this study. Another c.1006-3T>G variant at the same splice site has been previously reported as a likely pathogenic variant in an arrhythmia patient (van Lint et al., 2019). Although splicing analysis using RNA was not conducted in that study, *in silico* analysis using SpliceAI predicted an acceptor loss at position c.1006 for the c.1006-3T>G variant with a delta score of 0.45, similar to the predicted impact of the c.1006-44_1007delinsATTTTG variant identified in the present study.

The indel variant c.1006-44_1007delinsATTTTG is suggested to involve a functionally critical region because *RYR2* variants associated with CPVT are clustered within four mutational hotspots, including exons 3–15, 44–50, 83–90, and 93–105 (Steinberg et al., 2023). Although exon skipping could lead to either loss-of-function or gain-of-function processes depending on the structural consequences, the in-frame deletion induced by this variant was presumed to be associated with a gain-of-function mechanism and CPVT instead of being associated with a loss-of-function mechanism and CRDS. To date, the *RYR2* variants resulting in loss-of-function and CRDS have been predominantly located in the C-terminal cluster region (exons 83–90 and 93–105), and a few of the variants were located outside the mutational hotspots (Kallas et al., 2023; Steinberg et al., 2023). On the other hand, it is well known that deletion of the *RYR2* exon 3 located in the NTD preserves the reading frame and that it is associated with the CPVT-like phenotype (Steinberg et al., 2023). Exon 3 deletion induces conformational changes within the channel structure by disrupting interactions between the transmembrane domain and NTD, resulting in facilitated pore opening and calcium spillover (Lobo et al., 2011; Steinberg et al., 2023). In addition, from a structural perspective, the NTD comprises three subdomains, including NTD domain A (NTD-A), NTD domain B (NTD-B), and NTD domain C (NTD-C) (Hadiatullah et al., 2022). NTD-A and NTD-B contain 12 β strands that form a β trefoil domain. Three molecular mechanisms have been proposed for the NTD mutations: misfolding; destabilization of the interactions among NTD-A, NTD-B, and NTD-C; negative effects at the interfaces with the other *RYR2* domains (Hadiatullah et al., 2022). Exon 3 deletion removes the α helix and β4 strand of NTD-A but does not appear to cause severe misfolding (Lobo et al., 2011) (Figure 2E); instead, it is rescued by the flexible loop and affects the interfaces with other *RYR2* domains, including NTD-B. This change was proposed to induce the relative movements of the NTD, which is allosterically coupled to the pore region and confers a gain-of-function mechanism (Lobo et al., 2011). Similarly, exon 13 deletion observed in this study removes the β strands of NTD-B, which is expected to destabilize the intradomain interactions instead of misfolding (Figure 2D). Miotto et al. (2024) suggested that the mutant channels inducing local rotations of NTD-B weaken the intramolecular domain interactions and promote the primed state, which is the intermediate conformation between the closed and open states of wild-type RyR2 that is readily activated by stress stimulation. Superposition of the predicted protein structures of the NTDs of the wild type and exon 13 deletion showed milder rotation compared to exon 3 deletion (Figures 2D, E); this might explain the less-severe phenotype of the patients in this study than that of the RYR2 exon 3 deletion syndrome. By incorporating a phenotypic investigation of exercise-induced ventricular premature beats with the genotypic investigation of the *RYR2* indel variant, we classified the identified variant as likely pathogenic for CPVT.

Rucinski et al. (2020) demonstrated that CPVT patients had the highest rates of resuscitated sudden cardiac arrest compared to other cardiac inherited diseases. The probands often presented with more severe phenotypes than their genetically affected family members (Kallas et al., 2021). However, genetic surveillance of all familial members is recommended because genotype-positive but asymptomatic individuals can develop cardiac symptoms and may even require preventive treatment (Hayashi et al., 2009; Wilde et al., 2022). Genetically affected individuals should be evaluated for the resting ECG and exercise stress test. In this study, the proband’s mother had no history of syncope but showed frequent premature ventricular beats during the treadmill test. Consequently, the proband and her mother were successfully treated with the beta-adrenoreceptor blocker. Considering that the proband’s grandfather had no definitive risk factors for cardiovascular disease, he may have suffered cardiac arrest associated with CPVT; however, this could not be confirmed owing to lack of evidence (Figure 1A) as he had passed away more than 10 years ago and had never visited Samsung Medical Center or Samsung Changwon Hospital for treatment. For this reason, we were unable to evaluate his imaging test or medical records; our conclusions depend entirely on the statements of family members about his medical history, but they did not remember the finer details. Genetic surveillance for the *RYR2* indel variant was recommended to other asymptomatic family members (I:2 and II:1, Figure 1A); however, they have not undergone testing yet.

This study has some key limitations. First, the pathogenicity of the variant was speculated based on aggregating evidence of predicted structure changes by the *in silico* model and its potential effects on intramolecular domain interactions. This was not demonstrated by the experimental data of the protein structures, which may be different from the predictions. Specific experimental approaches (e.g., calcium release assays, patch-clamp electrophysiology, or RyR2 expression in heterologous systems) are therefore suggested as future work to validate the gain-of-function hypothesis. Second, the patients in this study were not evaluated through long-term follow-up. As *RYR2* exon 3 deletion syndrome shows complex CPVT-like phenotypes, including sinus node dysfunction, atrial tachycardia, and atrioventricular node conduction disorder (Steinberg et al., 2023), the patients may present with additional cardiovascular symptoms rather than those of CPVT in the future. Further studies are needed to investigate the disease mechanism and phenotypes of the *RYR2* indel variants for tailored treatment.

In conclusion, we investigated a single family manifesting CPVT and identified a novel *RYR2* indel variant with exon and intron involvement; further, we confirmed the pathogenicity of the variant. This study expands the mutational spectrum of *RYR2*-related CPVT. To interpret the *RYR2* variant appropriately, a comprehensive approach incorporating phenotypic and genotypic data should be considered.

## Data Availability

The data presented in the study have been submitted to the ClinVar repository, under Submission ID: SUB15372097.
