# Supplementary material for: Novel causative RYR2 indel variant with exon and intron involvement inducing exon 13 skipping in a family exhibiting catecholaminergic polymorphic ventricular tachycardia
Source: Front Genet. 2025 Jun 18;16:1581535. doi: 10.3389/fgene.2025.1581535 (PMC12213541; doi:10.3389/fgene.2025.1581535)
Supplement: Supplementary file 1 [file Table1.docx]

**Table S1.** Primer sequences used for amplification of the target region in this study.

| **Target** | | **Sequence (5’ → 3’)** | **Direction** | **Product size** |
| --- | --- | --- | --- | --- |
| *RYR2* DNA | Exon 13 | TGGCCATTATTTCAGGGGAC | F | 435 bp |
|  | Exon 13 | CAGTGGAATCTGCCCTTTCAC | R |  |
| *RYR2* cDNA | Exons 12-15 | GCTAAGAGTTGCGTGGAGTG | F | 578 bp |
|  | Exons 12-15 | GCTCTAAATGCTCATCTGGGG | R |  |

Abbreviations: F, forward; R, reverse.
